# Involvement of trimethylamine N-oxide in major depressive disorder via astrocytic d-Serine dysregulation

**DOI:** 10.1016/j.neurot.2026.e00909

**Published:** 2026-04-15

**Authors:** Liwang Lin, Longyu Li, Shiao Ren, Liping Liu, Ying Liu, Wenlei Zhang, Zengliang Gao, Xiaoting Ni, Haoxuan Li, Xinsheng Duan, Sijing Tao, Tianyu Zhao, Xin Hai

**Affiliations:** a Department of Pharmacy, First Affiliated Hospital of Harbin Medical University, 23 YouZheng Str, Nangang District, Harbin, China; b Clinical Laboratory of Psychiatry, First Psychiatric Hospital of Harbin, 217 Hongwei Road, Daowai District, Harbin, China; c Department of Pharmacology, College of Pharmacy, Harbin Medical University, 157 Health Care Road, Nangang District, Harbin, China

**Keywords:** Trimethylamine N-oxide, Major depressive disorder, Astrocyte, d-serine, AMPK/SIRT1

## Abstract

Trimethylamine N-oxide (TMAO), a co-metabolite of the gut microbiota and host, has been implicated in the pathogenesis of various neuropsychiatric disorders. However, its role in major depressive disorder (MDD) remains poorly understood. This study aims to verify the relationship between TMAO and MDD and elucidate the underlying mechanisms. Plasma TMAO concentrations were quantified by HPLC-MS/MS and compared between MDD patients and healthy controls. To explore the effects of TMAO on depressive-like behavior and cerebral d-serine metabolism, mice were administered TMAO via dietary supplementation. Finally, astrocytes were treated with TMAO to examine its impact on d-serine metabolism at the cellular level. Here, we found that plasma TMAO levels were significantly elevated in MDD patients and positively correlated with both HAMD-17 and HAMA-14 scores. Consistently, mice fed TMAO for seven weeks exhibited pronounced depressive-like behaviors. While TMAO exposure did not induce apparent astrocytic damage, it markedly promoted d-serine secretion and caused notable neuronal injury. Mechanistically, TMAO activated the AMPK/SIRT-1 signaling pathway in astrocytes, resulting in upregulated serine racemase expression. Furthermore, both exogenous d-serine and conditioned medium from TMAO-treated astrocytes triggered neuronal apoptosis. Collectively, these findings demonstrate that TMAO contributes to MDD pathogenesis by activating astrocytic AMPK/SIRT-1 signaling to enhance d-serine production, subsequently inducing neuronal apoptosis. TMAO may thus represent a promising therapeutic target for MDD.

## Introduction

Major depressive disorder (MDD), a significant psychiatric disorder, can cause great harm to individual physical and mental health [1]. MDD is influenced by a number of variables, such as environment and genetics [2], but stress is considered to be an essential risk factor for the onset of MDD [3]. Due to the lack of biomarkers that can be used for clinical diagnosis, the current diagnosis of MDD mainly relies on the subjective diagnosis of doctors [4,5]. Therefore, it is necessary to find a different understanding of MDD and new treatments.

Recent studies have found that trimethylamine N-oxide (TMAO), a metabolite dependent on the gut microbiota, has been linked to the emergence of several neurological and neuropsychiatric disorders [6,7]. TMAO is an endogenous substance primarily derived from dietary choline, carnitine, and related compounds. Notably, plasma TMAO has been shown to penetrate the blood-brain barrier (BBB) and accumulate in the brain [8] Preclinical studies have demonstrated that TMAO induces developmental defects in the central nervous system (CNS), triggers abnormal morphological and structural changes in neurons, and activates microglia and astrocytes [[9], [10], [11]]. However, plasma TMAO levels in patients with first-episode major depressive disorder (MDD) remain poorly characterized. Furthermore, the neurotoxic mechanisms underlying TMAO in the context of MDD are still inadequately understood.

Astrocytes, the largest and most abundant glial cells in the brain, play essential roles in neuroprotection, acid-base balance, blood-brain barrier formation, and maintaining homeostasis [12,13]. Previous investigations have demonstrated that astrocytes contribute to the pathogenesis of MDD, potentially through their aberrant activation [14]. Recent research has revealed that reactive astrocytes express serine racemase (SR), which catalyzes the conversion of l-serine to d-serine [15,16]. Under pathological conditions, excessive release of the excitatory neurotransmitters glutamate (Glu) and d-serine from hyperactive astrocytes can synergistically activate N-methyl-d-aspartate receptors (NMDARs) located on postsynaptic neuronal membranes, leading to a cascade of physiological and biochemical disruptions, neuronal injury, and ultimately excitotoxicity-induced cell death [15]. d-serine homeostasis in brain tissue is regulated not only by SR but also by 3-phosphoglycerate dehydrogenase (Phgdh) [17,18]. Disruption of any component in the d-serine synthetic pathway within astrocytes may result in dysregulated d-serine homeostasis, thereby potentially influencing the pathophysiology of MDD.

AMP-activated protein kinase (AMPK), a conserved serine/threonine kinase, modulates energy metabolism through the regulation of downstream molecular expression and activity. Sirtuin 1 (SIRT1), a critical downstream effector of the AMPK pathway, is intimately involved in diverse cellular physiological and biochemical processes. Emerging evidence indicates that TMAO can modulate AMPK/SIRT1 signaling [19]. Recent studies have demonstrated that the AMPK/SIRT1 pathway contributes to depression pathogenesis by influencing mitochondrial autophagy and cellular apoptosis [20]. Furthermore, AMPK/SIRT1 signaling has been implicated in serine biosynthesis [21].However, the precise mechanisms by which TMAO regulates AMPK/SIRT1 activity and subsequently affects d-serine biosynthesis in astrocytes remain elusive.

Thus, we conducted comprehensive validations at clinical, animal, and cellular levels to elucidate the relationship between the gut microbiota-derived metabolite TMAO and MDD, and to explore the potential mechanisms underlying TMAO-induced depressive pathogenesis.

## Methods

### Participants and plasma

The study involved 52 patients with major depressive disorders (MDD; median age: 30 years, 29 males), and 52 healthy controls (HC; median age: 30 years, 20 males). All participants were recruited between October 2019 and September 2022. MDD was diagnosed by psychiatrists based on the Mini-International Neuropsychiatric Interview (MINI) and DSM-IV [22]. And the severity of MDD was assessed using the Hamilton Depression Rating Scale-17 (HAMD-17) and Hamilton Anxiety Rating Scale-14 (HAMA-14). After fasting for 12 h, the elbow venous blood of all participants was withdrawn into a vacuum tube containing EDTA, then centrifuged at 4000 rpm, 4 °C for 5min, the plasma was separated and stored in the −80 °C freezer for TMAO measurement.

### Animals

Thirty-two four-week-old male C57BL/6J mice with specific pathogen-free (SPF) status were obtained from Liaoning Changsheng Biotechnology Co., Ltd. (Benxi, China). The mice were housed in a pathogen-free environment with a 12-h light/dark cycle, 50%–60% humidity, and ad libitum access to food and water. After one week of acclimatization, they were randomly divided into two groups: a control group receiving plain drinking water and a TMAO-fed group receiving water containing 1% TMAO for seven weeks. Cardiac function was assessed after feeding. All protocols adhered to Heilongjiang Laboratory Animal Management Regulations and the National Laboratory Animals Regulation, with approval from the Committee of Experimental Animal Care at the First Affiliated Hospital of Harbin Medical University (Harbin, Heilongjiang, China).

### Behavioral tests

After seven weeks of TMAO or control drink, six mice from each group were randomly chosen to undergo behavioral tests. The tests were conducted between 9 a.m. and 5 p.m. in a low-intensity light and sound-attenuation room, and were recorded by three individuals. Prior to the tests, the mice were acclimated to the room for at least 3 h. As previously mentioned, behavioral studies including tail suspension tests (TST), forced swimming tests (FST), sucrose preference tests (SPT), open-field test (OFT) and light/dark box were carried out [[23], [24], [25], [26]]. The methods of behavioral tests were presented in Supplementary Information.

### Measurement of TMAO and d-serine

TMAO concentrations were determined by high-performance liquid chromatography-tandem mass spectrometry (HPLC-MS/MS) using d9-TMAO (TRC, Toronto, Canada) as the internal standard according to a previous study [27]. The protein was precipitated by adding acetonitrile to plasma. 5 μL of sample supernatant was injected into the Agilent 1100 HPLC system (Agilent Technologies, CA, USA). The analytical method was presented in Supplementary Information and LC-MS/MS chromatograms of TMAO and d9-TMAO in plasma and brain were shown in Supplementary Figs. 1 and 2.

The sample was derivatized by Marfey's reagent before d-serine analysis. The derivatization process was given in the Supplementary Information. The products of d-serine derivatization were determined by HPLC-MS/MS using fudosteine (Aladdin, Shanghai, China) as an internal standard according to a previous study [28]. 5 μL of derivatization supernatant were injected into the 1100 HPLC system (Agilent Technologies, CA, USA). The analytical method was presented in Supplementary Information and LC-MS/MS chromatograms of L/d-serine and fudosteine in water, brain and extracellular medium were presented in Supplementary Figs. 3–5.

The accuracy and precision of these methods were tested. For TMAO, the accuracy was 95.6%∼103.2% and the precision 87.6%∼98%, For the product of L/d-serine derivatization, accuracies were 90.9%∼102.5% and 87.6%∼98%, respectively. And the precisions were 5.10% and 6.30%, respectively.

### Immunofluorescence staining

The frozen section procedure of brain was performed as described previously [29]. The sections were immunostained at 4 °C with a Glial fibrillary acidic protein (GFAP, 1:100, Abcam, Cambridge, USA) or postsynaptic density protein 95 (PSD95, 1:100, HUABIO, Hangzhou, China) antibody, then incubated with a secondary antibody (1:500 in PBS) at room temperature for 4 h. Then, DAPI (Diamidinophenyl indole, Beyotime, Shanghai, China) was used for nuclear staining (500 μg/mL stock; PBS for 1:500) for 10 min. A confocal microscope was used to take images. Reagents and methods are described in Supplementary Information.

### Cells culture and treatment

Cells were obtained from Institute of Neuroscience, Sino–Russian Medical Research Center, Harbin Medical University (Harbin, China) and grown in Dulbecco's modified Eagle medium (Thermo Scientific, Wilmington, USA) with 10% FBS (Beyotime, China) and 1% penicillin-streptomycin (Beyotime, China) in a humidified incubator at 37 °C filled with 5% CO_2_. Cells were maintained in a humidified incubator at 37 °C with 5% CO_2_. All experiments were performed using cells at passages 2–3. Treatments were applied by replacing the medium with fresh medium containing the indicated reagents, and cells were harvested at 90% confluence. TMAO was dissolved in serum-free DMEM according to the manufacturer's protocol. Astrocytes were subsequently treated with serum-free DMEM alone (0 μM TMAO) or TMAO at concentrations of 10, 20, 50, 100, and 200 μM for 24 h. Culture supernatants were then collected by centrifugation.

HT-22 cells were treated with 30 mM d-serine [30], 200 μM TMAO, 30 mM d-serine combine with 200 μM TMAO, astrocyte conditioned culture medium (ACM) and TMAO-exposed mixed astrocyte conditioned culture medium (TMAO-ACM) for 24h. For ACM and TMAO-ACM preparation, astrocytes were treated with serum-free DMEM alone or 200 μM TMAO for 24 h; the supernatants were then discarded, and fresh medium was added. After an additional 24 h, the conditioned media were collected. Neurons were cultured with conditioned medium at a 1:1 or 2:1 ratio with fresh medium.

### Cell-viability assay

Cell viability was assessed using the Cell Counting Kit-8 (CCK-8; Beyotime, Shanghai, China). Cells were seeded in 96-well plates at a density of 5000 cells per well and incubated for 24 h. Following drug treatment, cells were exposed to 10 μL CCK-8 solution for 1 h. Absorbance was measured at 450 nm using a microplate reader (BioTek, Vermont, USA).

### TUNEL assay

The TUNEL detection kit is utilized for DNA fragment labeling in order to identify cell death. TUNEL specifically stains DNA fragments, resulting in green fluorescence, while DAPI can bind to both intact DNA and fragments, resulting in blue fluorescence. Following drug treatment, cells were rinsed with PBS twice and then exposed to 100 μL of TUNEL detection solution for 60 min at 37 °C. Afterward, the cells underwent three subsequent PBS washes, DAPI staining for 5 min, and two additional PBS washes prior to observation under a fluorescence microscope.

### Western blotting and quantitative real-time PCR (qRT-PCR)

The methods and regents for western blotting and quantitative real-time PCR were described in the Supplementary Information. And the information about the primers was shown in the Supplementary Table 1.

### Bioinformatics analysis

The methods of bioinformatics analysis were according to a previous study by To et al. [31], the detailed experimental methods can be found in the supplementary materials.

### Statistical analysis

The relevant figure legends for each figure discuss the various statistical analyses that were performed for the different studies. The required sample size for animal studies was determined empirically based on the outcomes of prior research and was comparable to that frequently utilized in the field. Shapiro-Wilkes tests were used to assess normality in the distribution. Statistical analysis between two groups was tested using the Student's *t*-test or Mann-Whitney *U* test, where appropriate. The categorical data were expressed as numbers and percentages (%) and compared with the chi-square test. The correlation was measured using Pearson's or Spearman's correlation coefficients. Moreover, the logistic analysis with the diagnostic label of MDD or HC as the objective variables, and TMAO and others as explanatory variables was performed. Results were judged to be statistically significant if *P* < 0.05. Statistical analysis was performed using SPSS 24.0 software (IBM, NY, USA). Prism GraphPad 9.0 software (GraphPad Software, CA, USA) was used to generate the diagrams.

## Results

### Plasma TMAO concentrations in MDD were increased

**Fig. 1:**
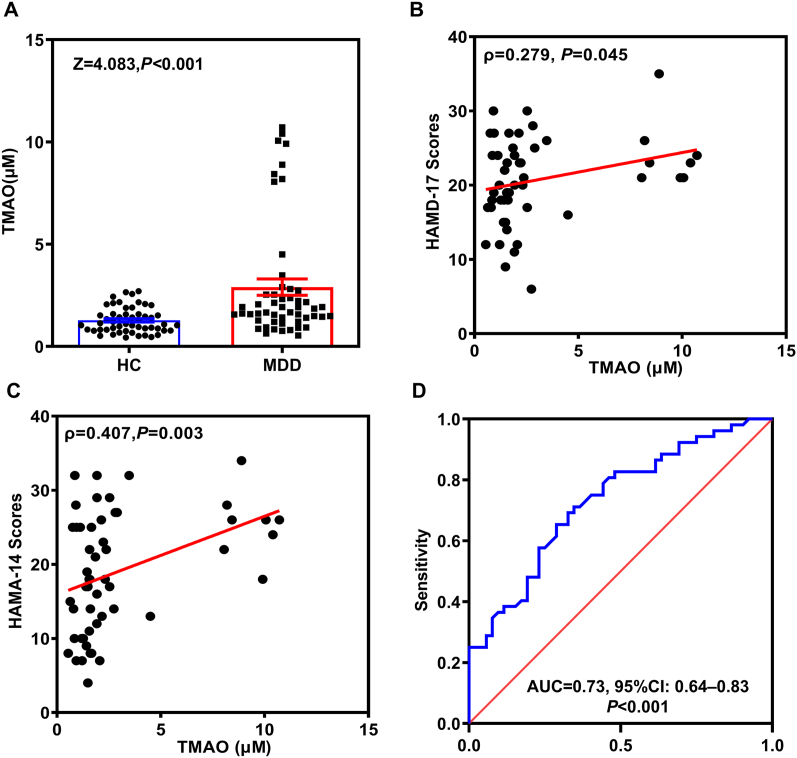
**The concentration of TMAO was increased in patients with MDD and could be used as a biomarker for the diagnosis of MDD.** A, plasma TMAO concentrations in patients with MDD compared with HC. B, Correlation between plasma TMAO concentrations and HAMD-17 scores in patients with MDD. C, Correlation between plasma TMAO concentrations and HAMA-14 scores in patients with MDD. D, ROC analysis of TMAO for predicting MDD. TMAO, trimethylamine N-oxide. HC, health control. MDD, major depressive disorder. ROC, receiver operating characteristic. Significant *P*-values are set at 0.05. N = 52 in each group.

To determine the association between TMAO and MDD, we measured plasma TMAO concentrations in healthy controls (HC) and patients with MDD. Table 1 summarizes the clinical characteristics of all participants. While baseline clinical variables showed no significant differences between groups, MDD patients exhibited a higher proportion of females. Notably, plasma TMAO levels were significantly elevated in MDD patients [median (IQR): 1.76 (1.30–2.69) μM] compared to HC [median (IQR): 1.09 (0.79–1.58) μM] (Fig. 1A).

**Table 1:** The clinical characteristics of the participants.

Variables	HC (52)	MDD (52)	*P* value
Age (years)	26 (23, 37)	27 (21, 38)	0.87^a^
Sex, male (%)	29, 55.8%	20, 38.5%	0.07^b^
BMI (kg/m^2^)	21.30 (19.54, 24.56)	22.10 (19.51, 25.08)	0.71^a^
Education (years)	16 (12, 16)	12 (9, 16)	**<0.001 ^a^**
Smoking (no/yes)	58/13	41/11	0.81^b^
HAMD-17 scores	2 (2, 2)	21 (17, 24)	**<0.001 ^a^**
HAMA-14 scores	0 (0, 2)	18 (12, 26)	**<0.001 ^a^**

Note: Data are presented as median (lower quartile, upper quartile) or percentage. Note: ^a^ Mann-Whitney *U* test. ^b^ Chi-squared test. Abbreviations: HC, healthy controls; MDD, major depressive disorder; HAMD-17, the 17-item version GRID Hamilton Depressive Rating Scale; HAMA-14, the 14-item version GRID Hamilton Anxiety Rating Scale. Significant *P*-values are shown in bold cases.

The correlations between plasma TMAO concentrations and clinical variables were summarized in Table 2. A positive correlation was observed between TMAO levels and smoking status in MDD patients (ρ = 0.28). Furthermore, we analyzed the associations between plasma TMAO concentrations and symptom severity scores in MDD patients. TMAO levels showed a significant positive correlation with HAMD-17 scores (Spearman's ρ = 0.28, Fig. 1B) and HAMA-14 scores (Spearman's ρ = 0.407, P = 0.003, Fig. 1C). Subsequently, multivariate logistic regression analysis was performed using diagnostic status as the dependent variable and TMAO, age, and other clinical factors as independent variables. The results revealed that each 1 μM increase in plasma TMAO concentration was associated with a 2.2-fold increased risk of MDD after adjusting for confounding clinical factors (Table 3).

**Table 2:** Correlations between plasma TMAO concentrations and the clinical variables.

	HC (52)		MDD (52)	
	ρ	*P*	ρ	*P*
Age (years)	0.16	0.25	−0.06	0.68
Sex, male (%)	−0.031	0.83	−0.16	0.26
BMI (kg/m^2^)	0.25	0.077	0.22	0.12
Education (years)	0.026	0.85	−0.098	0.49
Smoking (no/yes)	−0.14	0.31	**0.28**	**0.048**

Note: HC, healthy controls; MDD, major depressive disorder. ρ, Spearman's rank correlation coefficient. Significant *P*-values are shown in bold cases. ∗*P* < 0.05, ∗∗*P* < 0.01.

**Table 3:** The multiple logistic analysis with the diagnostic label of MDD or HC and clinical variables.

Variables	β	SE	*Wald*	*OR*	*P*
Age (years)	−0.023	0.028	0.70	0.98	0.40
Sex, male	0.81	0.52	2.40	2.24	0.12
BMI (kg/m^2^)	−0.023	0.055	0.18	0.98	0.67
Education (years)	−0.25	0.088	**8.32**	**0.78**	**0.004**
Smoking (no/yes)	−0.22	0.67	0.11	0.80	0.74
TMAO	0.399	0.16	**5.97**	**3.2**	**0.002**

Receiver operating characteristic (ROC) curve analysis was performed to evaluate the diagnostic performance of plasma TMAO concentrations for MDD. The optimal cutoff value for TMAO was determined to be 1.44 μM, yielding a sensitivity of 71.2% and specificity of 65.4% [AUC = 0.73, 95% CI: 0.64–0.83, Fig. 1D].

### TMAO supplementation caused depression-like and anxiety-like behavior in mice

**Fig. 2:**
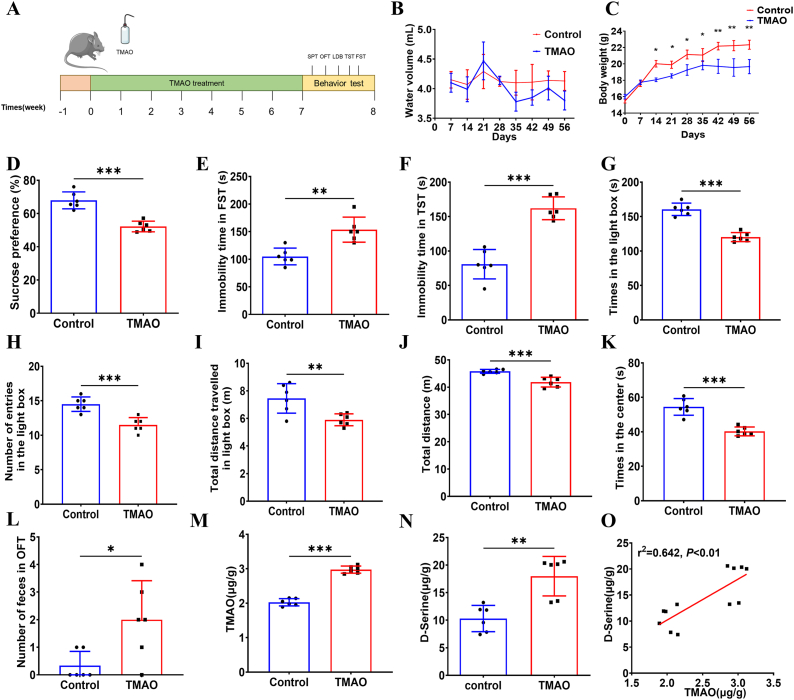
**TMAO treated mice developed depression-like behavior and anxiety-like behavior, and had increased TMAO and****d****-serine concentrations.** A, the schedule of TMAO-induced depression mouse model and behavioral tests. B, water intake from different groups of mice over 7 weeks. C, the body weight of the different groups. D, sucrose preference rates of the different groups. E, the immobility time in the forced swimming test (FST) of the different groups. F, the immobility time in the tail suspension test (TST). G, the time spent in the light box. H, the number of entries in the light box. I, the total distance in the light box. J, the total distance in open field test (OFT). K, the residence time in OFT. L, the number of feces of the different groups. M, TMAO concentrations in mouse brain tissues of the different groups. N, d-serine concentrations in mouse brain tissues of the different groups. O, correlation between brain TMAO concentrations and brain d-serine concentrations. All data are presented as mean ± SD. N = 6 each group. ∗ = *P* < 0.05, ∗∗ = *P* < 0.01, ∗∗∗ = *P* < 0.001.

Following the observation of elevated plasma TMAO levels in MDD patients, we established a mouse model of chronic TMAO exposure by administering 1% TMAO-supplemented drinking water to verify the causal role of TMAO in MDD pathogenesis (Fig. 2A). To ensure consistent TMAO intake, water consumption was monitored weekly at the same time point, with no significant difference observed between groups (Fig. 2B). Body weight, an indicator of depressive-like behavior, was also recorded weekly. Notably, TMAO-fed mice exhibited progressive body weight reduction compared to controls (Fig. 2C). After 7 weeks of TMAO administration, echocardiographic assessment was performed to exclude potential cardiac dysfunction that might confound locomotor activity. No significant differences were detected in left ventricular internal diameter at end-diastole (LVIDd), left ventricular internal diameter at end-systole (LVIDs), left ventricular ejection fraction (LVEF), or left ventricular fractional shortening (LVFS) between groups (Supplementary Fig. 6A–D). Additionally, the heart mass-to-body weight ratio showed no significant difference between control and TMAO-treated mice (Supplementary Fig. 6E), confirming that TMAO exposure did not impair cardiac function under these experimental conditions.

Subsequently, depressive-like behaviors were assessed using the sucrose preference test (SPT), tail suspension test (TST), and forced swimming test (FST). In the SPT, TMAO-fed mice exhibited significantly reduced sucrose preference compared to controls (52.18 ± 1.31% vs. 68.5 ± 2.34%, respectively; Fig. 2D), indicating anhedonia-like behavior. Furthermore, TMAO-treated mice displayed markedly increased immobility time in both the TST and FST compared to control mice (Fig. 2E and F), suggesting enhanced behavioral despair. Collectively, these results demonstrate that chronic TMAO exposure induces depressive-like phenotypes in mice.

Given the observed association between TMAO levels and anxiety symptom scores in MDD patients, we further evaluated anxiety-like behaviors in mice using the light-dark box test and open field test (OFT). In the light-dark box test, TMAO-fed mice exhibited significantly reduced time spent in the illuminated compartment and decreased locomotor distance in the white box compared to controls (Fig. 2G–I), indicating heightened anxiety-like behavior. Consistently, in the OFT, TMAO-treated mice displayed a marked reduction in total locomotor distance and central zone residence time, accompanied by a significant increase in fecal pellet output (Fig. 2J-L).

### The concentration of d-serine in brain tissue increased in mice supplemented with TMAO

To elucidate the mechanistic basis of TMAO-induced depressive-like behavior, we quantified TMAO and d-serine levels in whole-brain lysates using HPLC-MS/MS. Brain TMAO concentrations were significantly elevated in TMAO-fed mice compared to controls (Fig. 2M), indicating that TMAO efficiently crosses the blood-brain barrier. Notably, d-serine levels were also markedly increased in the brains of TMAO-treated mice relative to controls (Fig. 2N). Importantly, a strong positive correlation was observed between brain TMAO and d-serine concentrations (r^2^ = 0.852, Fig. 2O), suggesting that TMAO-mediated elevation of d-serine may represent a critical molecular mechanism underlying TMAO-induced depressive-like phenotypes.

### TMAO did not cause astrocyte damage but induces neuron injury

**Fig. 3:**
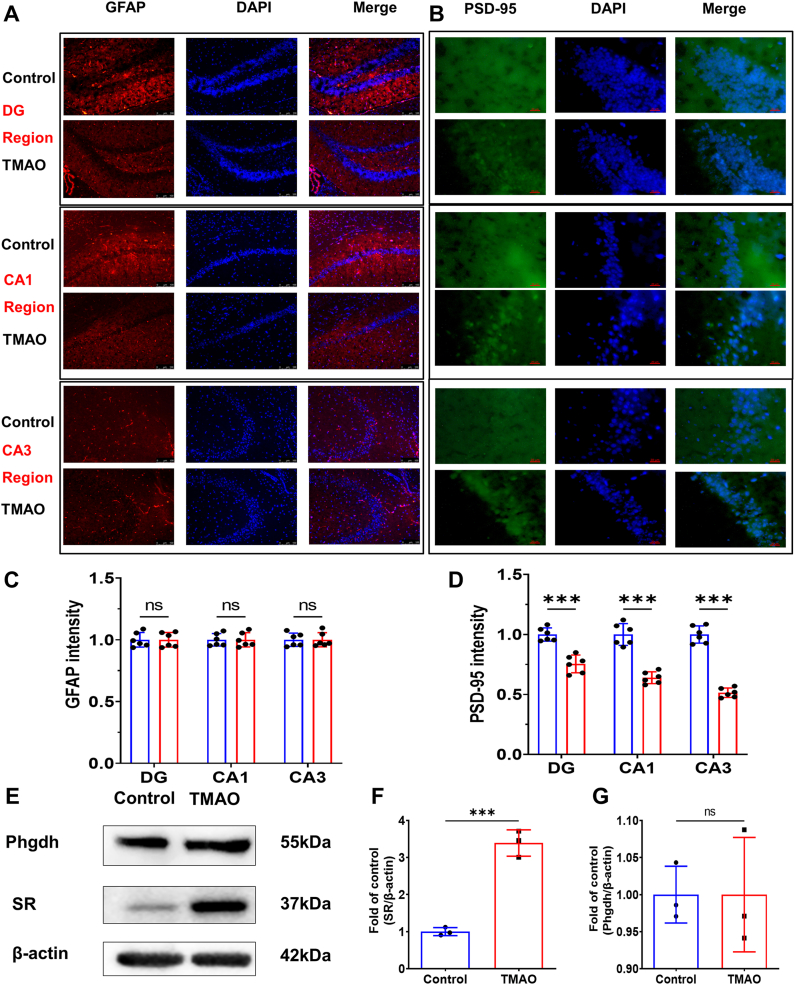
**The expression of SR was increased in the brain tissues of TMAO treated mice.** A, the GFAP results of hippocampal region in mice brain. B, the PSD-95 results of hippocampal region in mice brain. C, the GFAP intensity of hippocampal region. D, the PSD-95 intensity of hippocampal region. E, the representative Western blot results of Phgdh and SR. F&G, quantitative results of SR and Phgdh. All data are presented as mean ± SD. N = 3-6 each group. ∗ = *P* < 0.05, ∗∗ = *P* < 0.01, ∗∗∗ = *P* < 0.001.

To investigate the mechanism underlying TMAO-induced elevation of d-serine, we examined hippocampal astrocyte density and brain expression of d-serine racemase. Immunofluorescence staining was performed using GFAP (astrocyte marker) and PSD-95 (postsynaptic neuronal marker) in mouse hippocampal sections (Fig. 3A and B). Quantitative analysis revealed no significant difference in GFAP-positive astrocyte density/activation across distinct hippocampal subregions between TMAO-fed and control mice (Fig. 3C). However, a marked reduction in PSD-95-positive synaptic puncta density was observed in multiple hippocampal regions of TMAO-treated mice compared to controls (Fig. 3D).

### TMAO increased serine racemase expression in the brain

Having excluded astrocyte proliferation as a contributing factor, we next investigated d-serine synthetic machinery using Western blot analysis of brain tissue lysates (Fig. 3E). We first examined serine racemase (SR), the enzyme essential for d-serine biosynthesis. The results demonstrated significantly elevated SR protein levels in TMAO-treated mice compared to controls (Fig. 3F). Subsequently, we assessed phosphoglycerate dehydrogenase (Phgdh), the rate-limiting enzyme in l-serine synthesis and a prerequisite for d-serine production. Notably, no significant difference in Phgdh expression was detected between TMAO-fed and control mice (Fig. 3G), indicating that TMAO specifically upregulates SR-mediated d-serine racemization rather than de novo l-serine synthesis.

### TMAO did not cause astrocyte death but increased d-serine secretion

**Fig. 4:**
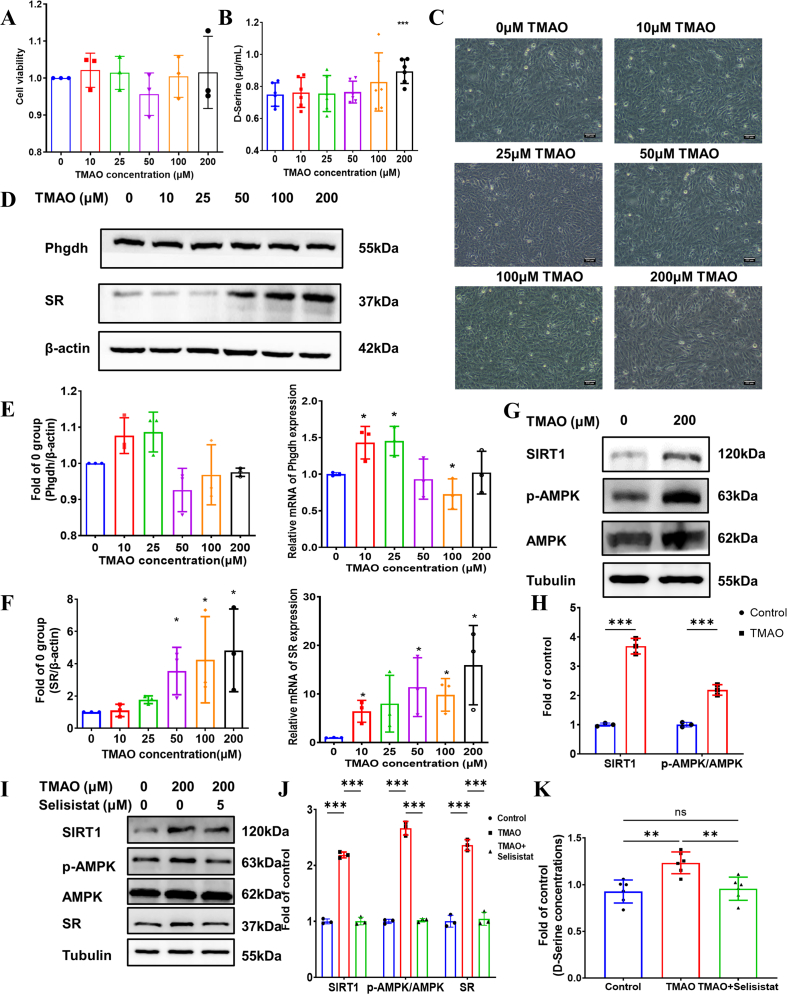
**TMAO did not cause astrocyte death, but it caused an increase in****d****-serine secretion and expression of SR.** A, AST viability after exposure to different concentrations of TMAO. B, d-serine concentration in cell culture medium. C, diagram of the cell under a light microscope. D&E, the Western blot images and quantitative results of Phgdh and SR. F, the mRNA levels of Phgdh and SR. G&H, the Western blot images and quantitative results of SIRT1 and p-AMPK/AMPK in control and TMAO groups. I–K, the Western blot images and quantitative results of SIRT1, p-AMPK/AMPK and SR in control, TMAO and TMAO combined with Selisistat groups. Gene expressions were normalized to GAPDH and presented as fold change vs the control group. TMAO, trimethylamine N-oxide. Phgdh, 3-phosphoglycerate dehydrogenase. SR, serine racemase. All data are presented as mean ± SD. Significant difference was defined as *P* < 0.05 using Student's *t*-test. ∗ = *P* < 0.05, ∗∗ = *P* < 0.01, ∗∗∗ = *P* < 0.001. N = 3 each group.

Reactive astrocytes represent a major source of pathological d-serine elevation in the central nervous system. To investigate the direct effects of TMAO on astrocytic function, primary astrocytes were treated with graded concentrations of TMAO (0, 10, 25, 50, 100, or 200 μM). Cell viability was assessed at 24 h using the CCK-8 assay, which revealed no significant cytotoxicity even at the highest concentration of 200 μM TMAO (Fig. 4A). These findings were corroborated by microscopic examination, which showed no overt morphological signs of cell death across all treatment groups (Fig. 4C). To determine whether TMAO directly stimulates astrocytic d-serine release, conditioned culture medium was collected after 24-h TMAO exposure and analyzed for d-serine content. Notably, treatment with 200 μM TMAO resulted in a significant increase in extracellular d-serine concentration compared to untreated controls (Fig. 4B), demonstrating that TMAO directly enhances astrocytic d-serine secretion in a cell-autonomous manner.

### TMAO increased the expression of SR in astrocytes

We subsequently investigated the molecular mechanisms underlying TMAO-induced d-serine synthesis and secretion in astrocytes. Western blot analysis revealed no significant alterations in Phgdh protein expression across all TMAO treatment groups (Fig. 4D and E). However, Phgdh mRNA levels exhibited a biphasic response: significant upregulation was observed at lower TMAO concentrations (10 and 25 μM), whereas 100 μM TMAO treatment resulted in decreased Phgdh transcription (P < 0.05; Fig. 4E ). In striking contrast, both SR mRNA and protein levels demonstrated a concentration-dependent increase with TMAO exposure, with 200 μM TMAO treatment producing the most pronounced elevation in SR expression (Fig. 4D-F). These results indicate that TMAO specifically upregulates SR at both transcriptional and translational levels, thereby enhancing the enzymatic conversion of l-serine to d-serine in astrocytes.

### TMAO increased the expression of SR by activating AMPK/SIRT1

**Fig. 5:**
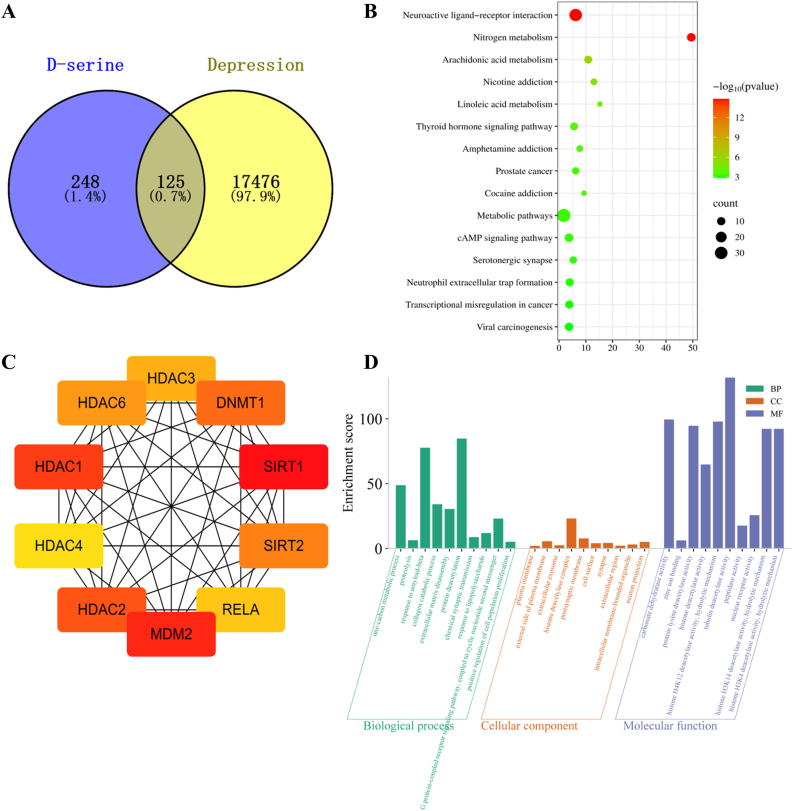
**Bioinformatics analysis of overlapping targets between****d****-serine and major depressive disorder.** A, overlapping targets between d-serine and major depressive disorder. B, network topology analysis of hub gene. C, KEGG pathway enrichment analysis of overlapping targets. D, GO functional analysis of overlapping targets.

SR serves as the rate-limiting enzyme in d-serine synthesis; however, its regulatory mechanisms remain largely elusive. A recent study demonstrated that AMPK could modulate gluconeogenic serine biosynthesis. To explore potential links between d-serine metabolism and depression, we performed a comparative analysis of d-serine-related targets and depression-associated genes, identifying 125 common targets (Fig. 5A). These targets were uploaded to the STRING database to construct a protein-protein interaction (PPI) network linking d-serine to major depressive disorder. Network analysis revealed that core targets with high degree values included SIRT1, HDAC4, and MDM2 (Fig. 5B). Subsequent GO and KEGG enrichment analyses indicated significant enrichment in neuroactive ligand-receptor interactions and one-carbon metabolic processes (Fig. 5C and D). Notably, among these core targets, SIRT1 is a well-established downstream effector of AMPK. Therefore, we investigated the involvement of the AMPK/SIRT1 pathway in TMAO-induced serine synthesis in astrocytes. Our results showed that 200 μM TMAO significantly upregulated the expression of p-AMPK/AMPK and SIRT1 (Fig. 4G and H). To further validate the role of SIRT1, astrocytes were co-treated with the SIRT1 inhibitor Selisistat and 200 μM TMAO. We found that Selisistat not only attenuated the phosphorylation of AMPK and reduced SIRT1 expression but also suppressed TMAO-induced upregulation of serine racemase (SR) (Fig. 4I and J). Consistently, co-treatment with Selisistat significantly decreased d-serine concentrations compared to TMAO treatment alone (Fig. 4K).

### TMAO-exposed astrocyte conditioned medium and d-serine induced neuron apoptosis

**Fig. 6:**
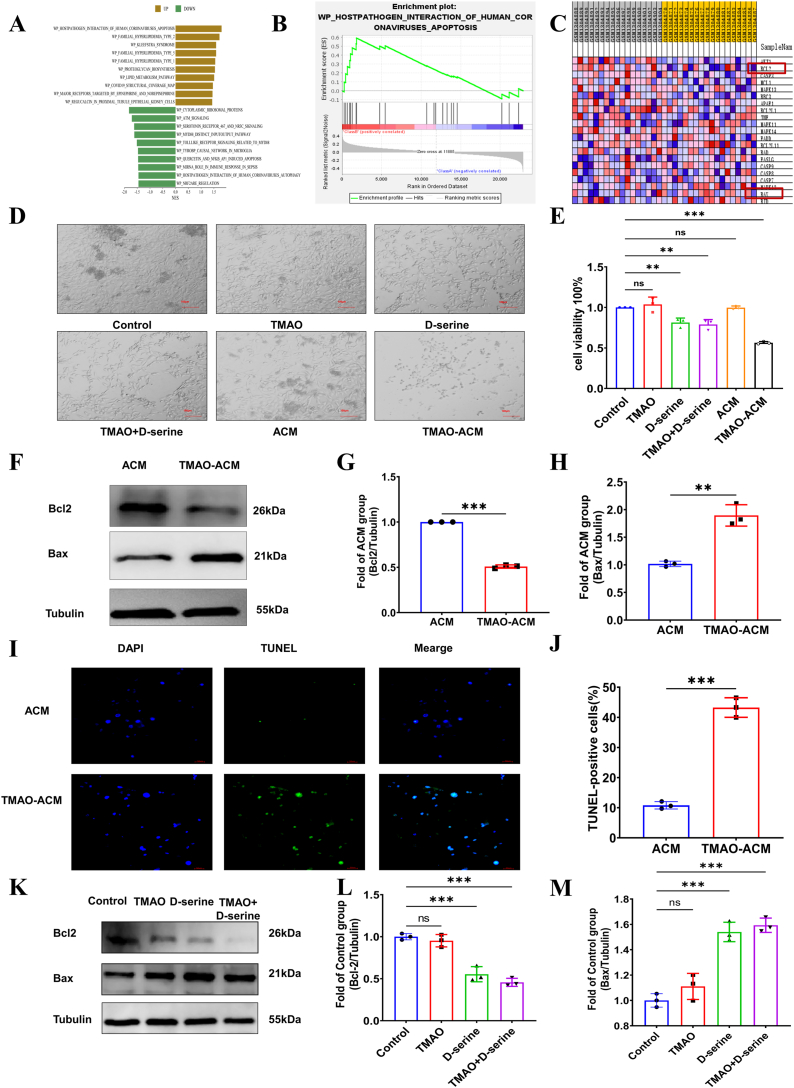
**TMAO-exposed astrocyte conditioned medium and****d****-serine induced neuron apoptosis.** A, the top ten differentially regulated signaling pathways from GSEA include both upregulated and downregulated pathways. B, GSEA revealed that depression upregulated the “WP HOSTPATHOGEN INTERACTION OF HUMAN CORONAVIRUSES APOPTOSIS” pathway expression. C, the heatmap of genes involved in the WP HOSTPATHOGEN INTERACTION OF HUMAN CORONAVIRUSES APOPTOSIS pathway. D, the cell white light diagrams of neuron after TMAO, d-serine and TMAO-ACM exposing. E, the CCK-8 of neuron after TMAO, d-serine and TMAO-ACM. F–H, the Western blot images and quantitative results of Bcl2 and Bax in ACM and TMAO-ACM groups. I&J, the Tunel assay of ACM and TMAO-ACM groups. K-M, the Western blot images and quantitative results of Bcl2 and Bax in control, TMAO, d-serine and TMAO- groups. ACM, astrocyte conditioned medium. TMAO-ACM, TMAO-exposed astrocyte conditioned medium. N = 3 each group. ∗ = *P* < 0.05, ∗∗ = *P* < 0.01, ∗∗∗ = *P* < 0.001.

GSEA analysis of depression-related transcriptomic data revealed significant enrichment of the "WP_Host-Pathogen Interaction of Human Coronaviruses_Apoptosis" pathway (Fig. 6A and B), with marked alterations observed in the anti-apoptotic protein Bcl-2 (Fig. 6C). Notably, neuronal apoptosis has been implicated in the pathogenesis of depression. To investigate the downstream effects of TMAO-induced astrocyte activation, we examined the impact of conditioned medium from TMAO-exposed astrocytes (TMAO-ACM) and exogenous d-serine on neuronal viability. Both bright-field microscopy and CCK-8 assays demonstrated that TMAO-ACM and d-serine treatment significantly induced neuronal death (Fig. 6D and E).

Subsequently, we investigated the impact of TMAO-ACM on neuronal apoptosis. Western blot analysis revealed that TMAO-ACM treatment significantly upregulated the pro-apoptotic protein Bax and downregulated the anti-apoptotic protein Bcl-2 (Fig. 6F–H). Consistently, TUNEL staining demonstrated a marked increase in TUNEL-positive cells in the TMAO-ACM group (Fig. 6I and J). To exclude the possibility of direct TMAO effects on neurons, we treated neurons with 200 μM TMAO or 30 mM d-serine independently. The results indicated that d-serine alone significantly increased Bax expression and decreased Bcl-2 expression, while TMAO appeared to exacerbate these pro-apoptotic effects (Fig. 6K-M).

## Discussion

Numerous research has demonstrated that the gut microbiota plays an important role in the development and progression of MDD [32]. However, current studies on the association between the gut microbiota derived metabolite TMAO and MDD are limited. In our study, plasma TMAO concentrations were measured in newly diagnosed MDD patients and healthy controls, and the results showed that plasma TMAO concentrations were higher in MDD patients than in HC. There were 8 (15%) of all MDD patients had the higher TMAO concentrations than the remaining 44 patients. And this phenomenon has also been observed in previous studies on TMAO [33]. Besides, as a metabolite dependent on gut microbiota, TMAO concentration is affected by age, gut microbiota composition, kidney function, and liver flavin monooxygenase [34]. Moreover, we found the plasma TMAO concentrations of MDD patients in our study were in accordance with the results of Liu et al. who analyzed plasma metabolites of depressed patients using ^1^H NMR-based metabolomics techniques. They found that plasma TMAO levels were significantly increased in depressed patients compared with controls [35].

It is noteworthy that approximately 15% of MDD patients in our cohort exhibited normal plasma TMAO levels, suggesting distinct endophenotypic heterogeneity within MDD. This observation aligns with emerging evidence that MDD comprises biologically distinct subtypes, including metabolically-driven and inflammation-driven endophenotypes [36]. TMAO elevation may specifically characterize a gut microbiota-dysbiosis-associated subtype of MDD, rather than representing a universal biomarker for all depressed patients. Future studies employing cluster analysis or stratification based on metabolic profiles are warranted to validate TMAO as a predictive biomarker for this specific MDD subset.

We subsequently assessed the correlation between plasma TMAO concentrations and HAMD-17 and HAMA-14 scores, which reflect the severity of depressive and anxiety symptoms, respectively. Our findings revealed a positive correlation between TMAO levels and both HAMD-17 and HAMA-14 scores. However, previous reports on the association between TMAO concentration and symptom severity remain limited. To our knowledge, only one study has demonstrated that serum TMAO concentrations in depression patients with carbohydrate malabsorption (CMA) were positively correlated with depressive symptom severity [37]. Besides, we made a logistic regression**.** The results indicated that each 1 μM increase in TMAO concentration was associated with a 2.2-fold increased risk of depression. TMAO concentration is associated with the occurrence and severity of MDD.

The ROC curve analysis was performed to evaluate the diagnostic value of plasma TMAO. The results indicated an optimal cutoff value of 1.44 μM (AUC = 0.732, 95% CI: 0.637–0.828) for distinguishing MDD patients from healthy controls (HC). These findings suggest that TMAO possesses potential diagnostic value. However, TMAO concentrations exceeding this threshold do not necessarily indicate MDD. Rather, elevated TMAO levels may serve as a trait marker rather than a state marker of MDD.

Accumulating evidence suggests that microbial metabolites may mediate the relationship between environmental factors and behavior, as well as central nervous system function [9]. To determine whether TMAO acts as a causative agent in MDD, mice were administered TMAO via dietary supplementation, and behavioral phenotypes were subsequently assessed. The results revealed that TMAO-fed mice exhibited a range of depressive- and anxiety-like behaviors compared to control mice. These findings are consistent with those of Luo et al. who reported that TMAO exposure significantly affects depression-, anxiety-, and cognitive-related behaviors in mice [6]. Collectively, these results indicate that TMAO may serve as a causal factor in MDD pathogenesis, providing novel insights into the impact of microbial metabolites on psychiatric disorders.

To elucidate the mechanisms underlying TMAO-induced MDD, we conducted both animal and cellular studies. Following dietary TMAO administration, we observed a concurrent increase in brain tissue TMAO concentrations, indicating that TMAO crosses the blood-brain barrier. These findings are consistent with previous reports demonstrating that TMAO can penetrate the blood-brain barrier. [38,39]. When TMAO penetrates the brain, it could firstly come into contact with astrocytes. In the current study, we observed the effect of TMAO on astrocytes in the mouse hippocampus using GFAP, and the results showed that TMAO did not decrease the number of astrocytes. Various TMAO concentrations (0-200 μM) were used to treat astrocytes, but no obvious cell death was observed. This was consistent with the results of a study on TMAO and brain tissue aging, which found astrocytes treated with 100 μM TMAO did not cause astrocyte death [11]. We should focus on the effects of TMAO on astrocytes function.

Li et al. reported that activated astrocytes exhibit altered serine racemase (SR) expression and d-serine content [16]. d-serine is a potent co-agonist of N-methyl-d-aspartate receptors (NMDARs), involved in NMDAR-mediated neurodevelopment and neurotransmission [40]. While Hashimoto et al. found elevated serum d-serine concentrations in patients with MDD [41], Mitani et al. reported no significant differences in plasma L- and d-serine levels between MDD patients and healthy controls [42]. In the present study, we found that d-serine concentrations were increased in the brain tissue of TMAO-fed mice and that TMAO treatment enhanced d-serine secretion from astrocytes. These findings are consistent with those of Ahnaou et al. who demonstrated that elevated brain serine concentrations induce depression-like behaviors in mice [43]. Collectively, these results suggest that TMAO may contribute to MDD pathogenesis by promoting d-serine production, implicating TMAO as a potential pathogenic factor in depression.

Notably, the observed increase in d-serine concentration was not attributable to astrocyte proliferation. We subsequently examined enzymes involved in d-serine synthesis as well as transporters mediating d-serine uptake and release. SR, the key enzyme catalyzing the conversion of l-serine to d-serine in the brain [44], was significantly upregulated following TMAO administration in both animal and cellular models. Moreover, TMAO treatment increased SR expression in astrocytes in a dose-dependent manner. l-serine, the precursor of d-serine, also plays an essential role in d-serine synthesis. In resting astrocytes, l-serine is synthesized from glucose via the pentose phosphate pathway under the catalytic action of Phgdh. [18]. Our study showed no significant changes in Phgdh expression, suggesting that TMAO selectively affects d-serine synthesis without altering l-serine production. These results indicate that TMAO increases d-serine concentrations by upregulating SR expression, thereby contributing to MDD pathogenesis.

SR is the key enzyme catalyzing d-serine synthesis. To elucidate the mechanism underlying TMAO-mediated SR upregulation, we investigated the AMPK signaling pathway. AMPK functions as a cellular energy sensor, monitoring intracellular energy status through the AMP-to-ATP ratio [45]. It plays a pivotal role in regulating cellular processes under both energy-deficient and energy-excess conditions. Upon activation, AMPK promotes energy production while suppressing energy consumption [46]. A study on d-serine synthesis found that AMPK activation significantly increases serine production [21]. Based on these findings, we hypothesized that TMAO regulates SR expression through the AMPK/SIRT1 signaling pathway in astrocytes. Our experiments revealed that TMAO exposure increased the expression of phosphorylated AMPK (p-AMPK) and SIRT1 in astrocytes. Subsequent co-treatment with the SIRT1 inhibitor Selisistat reversed these effects, resulting in decreased p-AMPK, SIRT1, and SR expression, as well as reduced d-serine concentrations.

The observation that Selisistat (SIRT1 inhibitor) attenuates p-AMPK levels suggests potential reciprocal regulation or feedback loops between AMPK and SIRT1, consistent with literature reports of bidirectional AMPK-SIRT1 crosstalk [47]. While our data demonstrate that TMAO enhances both AMPK phosphorylation and SIRT1 activity, we cannot conclusively establish unidirectional causality. AMPK may activate SIRT1 through NAD + metabolism, while SIRT1-mediated deacetylation of LKB1 could feedback to modulate AMPK activity. Future studies employing specific AMPK activators/inhibitors in SIRT1-knockdown cells (and vice versa) will be necessary to dissect the precise directional relationship in TMAO-stimulated astrocytes. These results suggest that the AMPK/SIRT1 pathway is involved in TMAO-mediated SR regulation. However, our findings appear inconsistent with previous reports. A recent study demonstrated that TMAO inhibits the AMPK/SIRT1 pathway, thereby promoting inflammation in vascular cell models [19]. This discrepancy may be attributed to differences in cell types or experimental models employed.

Moreover, excessive d-serine synthesis and release can induce NMDAR overexpression and aberrant signal transduction in neurons, which are considered early indicators of neuronal injury [48]. A previous study demonstrated that lanthanum, a metallic element, enhances serine synthesis in astrocytes, subsequently inducing neuronal excitotoxicity [49]. Our study demonstrates that TMAO increases d-serine concentrations and induces neuronal damage in mouse brain tissue. Furthermore, treatment with TMAO-exposed astrocyte-conditioned medium significantly enhances neuronal apoptosis. These results indicate that TMAO can trigger neuronal excitotoxicity, potentially mediated by excessive d-serine release from hyperactive astrocytes leading to NMDAR overactivation. Notably, while exogenous d-serine alone significantly increased neuronal apoptosis, TMAO alone did not induce significant neuronal death, suggesting that astrocyte-derived d-serine is the primary mediator of TMAO-associated excitotoxicity. This finding aligns with the study by Perez et al. who reported that enhanced astrocytic d-serine production and release following controlled cortical impact injury contributes to synaptic damage and functional impairment [15].

**Fig. 7:**
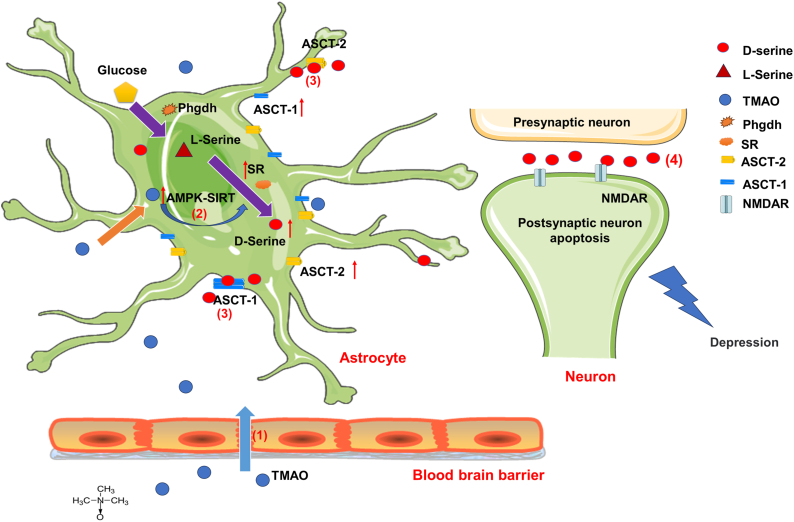
The proposed mechanisms of TMAO increased d-serine and influenced the neuron.

The proposed mechanisms underlying TMAO-induced MDD are illustrated in Fig. 7. This study demonstrates that elevated circulating TMAO crosses the blood-brain barrier and accumulates in brain tissue. TMAO subsequently activates the AMPK/SIRT1 signaling pathway in astrocytes, leading to upregulated SR expression and enhanced d-serine synthesis and release. The resulting elevation in extracellular d-serine concentrations promotes NMDAR overactivation, ultimately triggering neuronal apoptosis and contributing to MDD pathogenesis. However, this study has several limitations. The clinical sample size was relatively small, and future studies should incorporate larger, multi-center cohorts to validate these findings.

## Author contributions

Liwang Lin: Writing – original draft, Methodology, Formal analysis. Longyu Li: Writing – original draft, Methodology, Formal analysis, Data curation. Shiao Ren: Writing – original draft, Methodology, Formal analysis. Liping Liu: Writing – review & editin, Resources. Ying Liu: Writing – review & editing, Validation. Wenlei Zhang: Writing – review & editing, Validation. Zengliang Gao: Writing – review & editing, Validation. Xiaoting Ni: Writing – review & editing, Validation. Haoxuan Li: Writing 0– review & editing, Validation. Xinsheng Duan: Writing – review & editing, Validation. Sijing Tao: Writing – review & editing, Validation. Tianyu Zhao:Writing – review & editing, Funding acquisition. Xin Hai: Writing – review & editing, Supervision, Methodology, Funding acquisition, Conceptualization.

## Declaration of competing interest

The authors declare that they have no known competing financial interests or personal relationships that could have appeared to influence the work reported in this paper.
