# Supplementary material for: Involvement of trimethylamine N-oxide in major depressive disorder via astrocytic d-Serine dysregulation
Source: Neurotherapeutics. 2026 Apr 15;23(3):e00909. doi: 10.1016/j.neurot.2026.e00909 (PMC13096918; doi:10.1016/j.neurot.2026.e00909)
Supplement: Multimedia component 1 [file mmc1.docx]

**The gut microbiota derived metabolite trimethylamine N-oxide (TMAO) is linked to major depressive disorder, with D-serine playing a crucial role**

**Journal name: brain, behavior and immunity**

**Supporting information**

**Materials and methods**

**Inclusion and Exclusion Criteria**

MDD was diagnosed by psychiatrists based on the Mini-International Neuropsychiatric Interview. The DSM-IV (American Psychiatric Association, 2000) criteria were used to evaluate patients with MDD.

Inclusion criteria for MDD: (1) The total score of the 17-item Hamilton Depression Scale (HAMD) is ≥7 at baseline; the 14-item Hamilton Anxiety Scale (HAMA) is ≥7 at baseline; (2) was first diagnosed and had never taken antipsychotic drugs; (3) male or female aged 16–60 years; (4) written informed consent was obtained from patients; (5) there was no significant change in dietary pattern. Exclusion criteria for MDD: (1) Suffering from serious physical or mental illness; (2) suffering from chronic diseases such as diabetes and hypertension; (3) previous history of psychoactive substance abuse (except tobacco and alcohol); (4) pregnant or lactating women; (5) any possible or drugs impact biomarkers: long-term regular use of NSAIDs, COX-2 inhibitors, immunosuppressants, hormonal drugs, interferon, chemotherapy drugs, anticoagulants, etc.

Inclusion criteria for healthy controls: (1) Voluntarily participated in the study, informed of the contents of the experiment and signed the written informed consent; (2) male or female aged 16-60 years; (3) no previous or current diagnosis of any mental disorder; (4) not participating in any drug experiment in the past 6 months. Exclusion criteria for healthy controls: (1) Suffering from serious physical or mental illness; (2) suffering from chronic diseases such as diabetes and hypertension; (3) previous history of psychoactive substance abuse (except tobacco and alcohol); (4) pregnant or lactating women; (5) any possible or drugs impact biomarkers: long-term regular use of NSAIDs, COX-2 inhibitors, immunosuppressants, hormonal drugs, interferon, chemotherapy drugs, anticoagulants, etc.

**Echocardiography**

The mouse heart was detected by ultrasound using a small animal ultrasound imaging system Vevo2100 sonograph (VisualSonics, Canada). First, the mice were anesthetized. After complete anesthesia, the hair from the chest to the upper abdomen was removed with depilating cream, and the mice were placed on the ultrasound panel in a supine position. The limbs of the mice were secured to the ultrasound electrode piece using tape, and a coupler was applied to the skin of the limbs as well as the heart region to avoid air bubbles.

By echocardiography, a 30 MHz imaging sensor system for testing, in the detection of long axis, the ultrasonic probe incisure towards the mouse head, counterclockwise around 45 °, adjusts the probe to the skin nicely with mice. After opening the b-type ultrasonic mode according to the image, adjust the operation panel until it can be clearly observed in the left ventricular long axis type B ultrasonic cardiogram. Then, the M-mode echocardiography was turned on, the sampling line was adjusted to the correct position, and the M-mode echocardiography was pressed again to obtain the left ventricular long axis M-mode echocardiography. Detection of the short axis will probe the incisure clockwise around 90°, according to the screen image, to adjust the ultrasonic operation panel until it shows clear with two circular cardiograms of mastoid muscle. The procedure described above was repeated to obtain B-mode and M-mode echocardiograms.

The left ventricular ejection fraction (EF%), left ventricular fractional shortening (FS%), left ventricular end-diastolic diameter (LVIDd) and left ventricular end-systolic diameter (LVIDs) were measured by echocardiography.

**The methods of Behavioural tests**

**Sucrose preference test (SPT)**: This test is used to evaluate depression-like behavior in laboratory animals based on the characteristics of mice's preferences for sweet taste. In the experiment, the mice were first acclimated in a quiet room for two days, and the sucrose was dissolved in sterile water and configured with a concentration of 1% sucrose solution. Then sterile water and a 1% sucrose solution were put into drinking water bottles and placed together in the drinking place of the mouse cage. 12 hours to change positions. The sugar-water preference test was carried out immediately after adaptation. During the test, water solution and sucrose solution were also placed in one bottle per cage and exchanged for 12 hours. Sucrose solution, ordinary drinking water, and total consumption were obtained by calculating the 24-hour change in drinking bottle quality and assessed using the sugar water preference index.

**Forced-swim test (FST)**: Mice were placed in a clear, transparent glass container, and the experiment was conducted using a 2 L beaker. Add warm water at 23–25 °C to the beaker so that the height of the water reaches 20 cm. Mice were put into it to swim. Each mouse had a total of 6 minutes: the first 2 minutes of adaptation and the total time of inactivity in the last 4 minutes were recorded by three different observers, and the average was calculated. The mice were judged to be stationary when they stopped struggling, floated in the water, or made only small movements of their limbs to keep their heads above water.

**Tail suspension experiment (TST)**: The mice were placed in a quiet room for 3 hours before the experiment began. First of all, prepare an iron rack with iron bars, and then glue the medical tape to the mouse tail 2 cm from the end of the end, so that it is upside down hanging on the iron bar and the mouse head is about 15-20 cm off the ground. Each mouse had a total of 6 minutes of adaptation. And then the total time of inactivity for the next 4 minutes was recorded by three different individual observers, and the average was calculated. The immobile state was based on the standard that the mouse gave up the struggle and the body remained in a static, overhanging state.

**Open-field test (OFT)**: Mice were placed into the center of a dimly lit (20–30 lux) chamber of the open-field apparatus (86×86×50). Movements of the animals were tracked by an automatic monitoring system (Any-maze, Stoelting, USA) for six minutes. Horizontal motor activity was evaluated by calculating the distance that the animals travelled in the arena. For each group, the mean value and SEM was calculated.

**Light/dark test:** The apparatus consists of two compartments: an open and illuminated ‘light’ compartment and an enclosed ‘dark’ compartment (see Figure 1C). The compartments are typically connected by a small opening located in the center of the apparatus; however, modified versions of the task include a tunnel connecting the two chambers. Test sessions are typically six minutes in duration and are conducted under varying degrees of white light; increasing or decreasing light intensity can decrease or increase, respectively, the expression of risk assessment behavior and the duration of time spent exploring the white compartment. The aversive nature of the light/dark test is based on rodents’ innate aversion to two mild stressors: the white light, and the novel and potentially threatening environment. Primary measures of anxiety-like behavior include the percentage of time spent in the light vs. dark compartments, the number of light entries, latency to enter the light compartment and risk assessment.

**Chromatographic and mass spectrometry conditions for TMAO measurement.**

5 μL of sample supernatant were separated by chromatography at a flow rate of 0.4 mL/min on a Luna Silica column (3 µm, 100 mm🞨2 mm, Phenomenex) at 25°C. 75% acetonitrile with 25% water containing 5 mM ammonium formate and 0.1% formic acid (v/v/v, Solution-A) and acetonitrile (Solution-B) were employed as mobile phases. Solution-A: Solution-B = 3: 2. We monitored TMAO and its internal standard, d9-TMAO, using an API 4500 triple quadrupole mass spectrometer (AB Sciex, CA, USA) in positive mode with characteristic parent to daughter ion transitions: m/z 76 → 58 for TMAO and m/z 85 → 66, respectively. The LC-MS/MS chromatograms of TMAO and d9-TMAO are presented in Supplementary Figure 1 and 2.

**The method of derivatization**

100 μL brain tissue homogenates were added with 20μL NaHCO3 (200mg/mL), 20uL Fudosteine (50μg/mL), and 40μL Marfey reagent (1mg/mL). After vortexing and mixing, the reaction was carried out at 60 °C for 2 h. At the end of the reaction, 20μL of formic acid solution was added to terminate the reaction, and finally acetonitrile was added to precipitate the protein. The supernatant was taken for detection.

**Chromatographic and mass spectrometry conditions for D-serine measurement.**

The chromatographic separations of analytes were performed on a C18 column (3 µm, 100 mm🞨2 mm, Waters) with a flow rate of 0.4 mL/min at 25°C. A linear gradient of Solution-A (water containing 10 mM ammonium acetate and 0.1% formic acid, v/v) and Solution-B (methanol) was delivered using the following profile (min/% MP B): 0.0–9.0/45, 9.0–9.5/45–80, 9.5–16.5/80, 16.5–17.0/80–45 and 17.0–20.0/4. Characteristic production transitions used for quantitation were m/z 356→162.1 for D-serine and m/z 430→91 for fudosteine. The LC-MS/MS chromatograms of serine and fudosteine are presented in Supplementary Figure 3-5.

**The methods of Western Blot**

RIPA lysis buffer (Beyotime, China) containing a protease inhibitor (Beyotime, China) was used to lyse samples of mouse brain tissue and CTX TNA2 cells. We measured the protein concentration using the BCA Protein Assay Kit (Beyotime, China). Protein was loaded onto 10% polyacrylamide gels (EpiZyme, China) and then transferred to polyvinylidene difluoride membranes (Roche, Basel, Switzerland). The membranes were incubated with primary antibodies against SR (1:1000; ABclonal Technology, China, A21158), Phgdh (1:1000; ABclonal Technology, China, A10461), BCL-2 (1:1000; ABclonal Technology, China, A19693), BAX (1:1000; ABclonal Technology, China, A20227), SIRT1 (1:1000; ABclonal Technology, China, A11267), AMPK (1:1000; ABclonal Technology, China, AP1441), p-AMPK (1:1000; MCE Technology, China, HY-P80451), Tubulin and β-actin (1:2000; Zhong Shan -Golden Bridge, China) at 4℃ overnight after blocking with 5% skim milk for 60 mins. After that, the membranes were incubated with secondary antibodies (1:5000; Wuhan SanYing, China) at 25℃ for 60 minutes. Gel imaging and a chemiluminescence imaging system were used for densitometric analysis (CLINX, China). Fold changes adjusted to control group values were used to report the results.

**The methods of Quantitative real-time PCR (qRT-PCR)**

The TRIzol reagent (Invitrogen, CA, USA) was used to extract total RNA from CTX TNA2 cells in a fume hood. The cDNAs were generated from the total RNA using a reverse transcription kit (Seven, Beijing, China) according to the instruction. Gene expressions were compared to the level of GAPDH. SYBR Green Master Kit (GeneBatter, China) was used to perform real-time PCR on the SLAN-96S (Hongshi, China). The Ct technique was used to determine the relative mRNA expression. In Supplementary Table 1, the primer sequences listed below were shown.

**Supplementary Table1. Primer sequences used for qPCR.**

| Gene | Forward 5′–3′ | Reverse 5′–3′ |
| --- | --- | --- |
| SR | CAGAGGTGTAACGCTGGGAAG | GCAAAGGGTGTAAGGTCATTTAGG |
| ASCT1  ASCT2  PHGDH  GAPDH | AGGCAACAATGGTGTTGGTGTC  CCACATGCGAAAAGGAATCT  TGGCAGAAGCATTGGGCACAC  CAATGTGTCCGTCGTGGATCT | ATGGCCCAGTATAGAGGCGAAG  CTCAAGAGCCCAATTTCCAA  GCAGGTCCCAGCATTCTTCAGAG  GTCCTCAGTGTAGCCCAAGATG |

**Bioinformatics analysis**

To elucidate the connections among the intersecting target genes involved in TAX's therapeutic effects against cardiotoxicity, a protein-protein interaction (PPI) network was generated using the STRING database (species: Homo sapiens) (https://string-db.org/). The network relationships of target interactions were obtained, and the TSV format file was downloaded for import into Cytoscape 3.9.1 software for visualization and topological analysis. Subsequently, the CytoHubba plugin was utilized to analyze and identify the top ten target genes based on degree centrality ranking. Using GSEA software, the association of genes with the phenotype (presence or absence of MDD) was quantified by calculating the NES values, which were then ranked in descending order. Subsequently, for each annotated gene within the gene set, we assessed whether it was enriched in the upper or lower portions of the ranked gene list, thereby determining the impact of gene co-variation within the set on phenotypic changes.


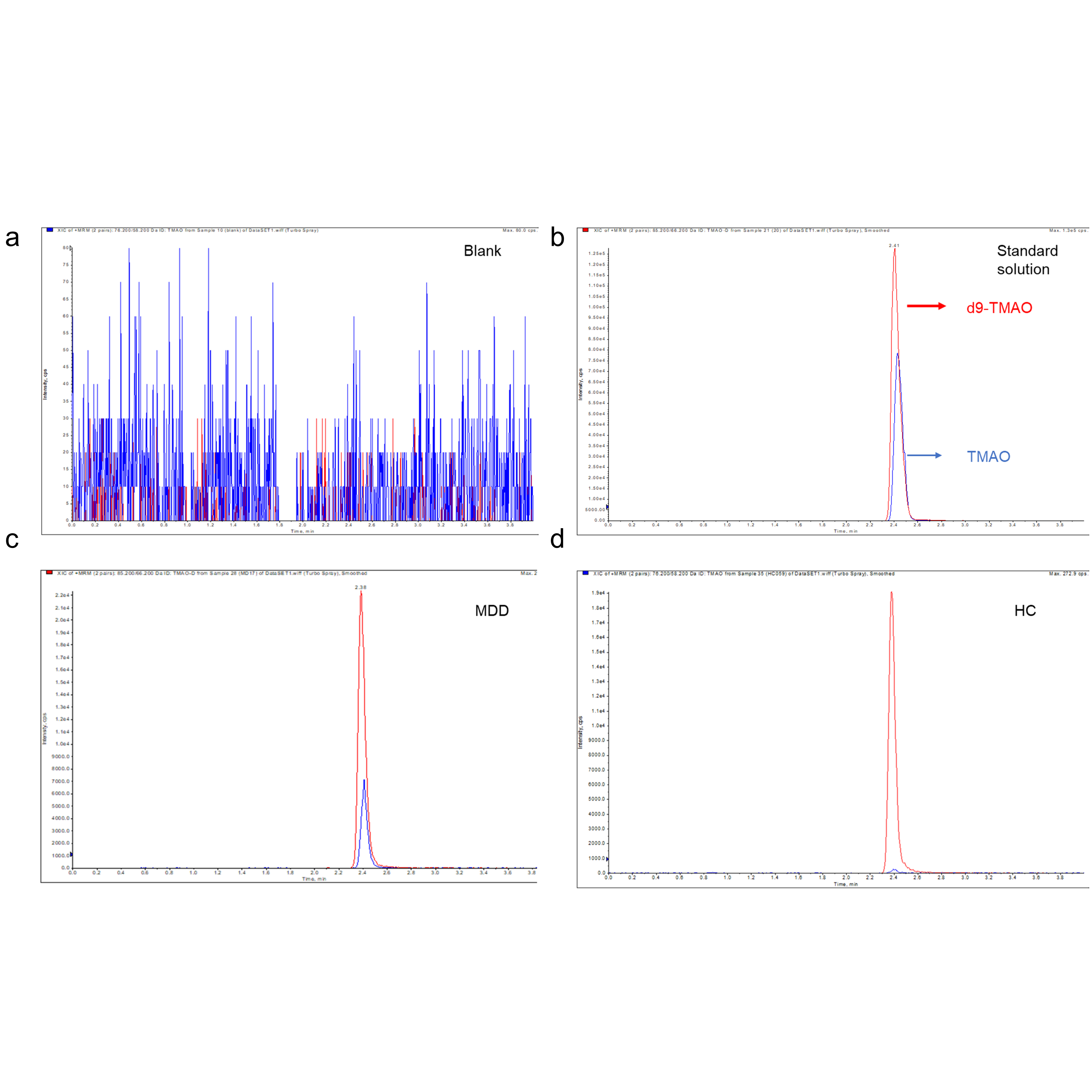


**Supplementary Fig. 1 Representative LC-MS/MS chromatograms for TMAO in plasma.** a, chromatogram of the blank plasma sample; b, chromatogram of the blank plasma sample with the standard 10 μmol/L TMAO and 50 μmol/L d9-TMAO; c, chromatogram of the plasma sample of HC; d, chromatogram of the plasma sample of MDD. TMAO, trimethylamine N-oxide; HC healthy control; MDD, major depressive disorder. Blue lines represented TMAO and red lines represented d9-TMAO.


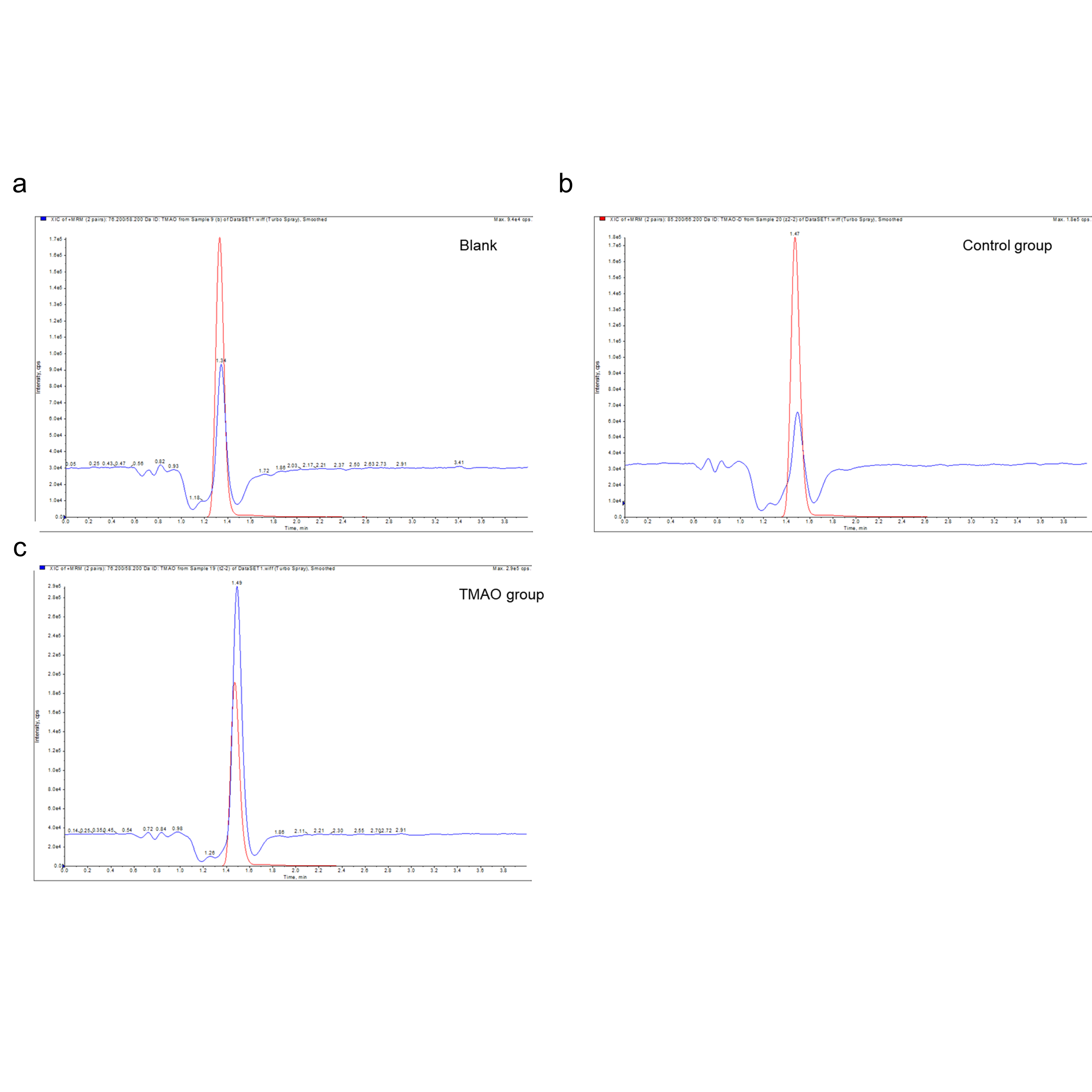


**Supplementary Fig. 2 Representative LC-MS/MS chromatograms for TMAO in brain.** A, chromatogram of the blank brain sample; B, chromatogram of the brain sample in control group; C, chromatogram of the brain sample in TMAO fed group. TMAO, trimethylamine N-oxide. Blue lines represented TMAO and red lines represented d9-TMAO.


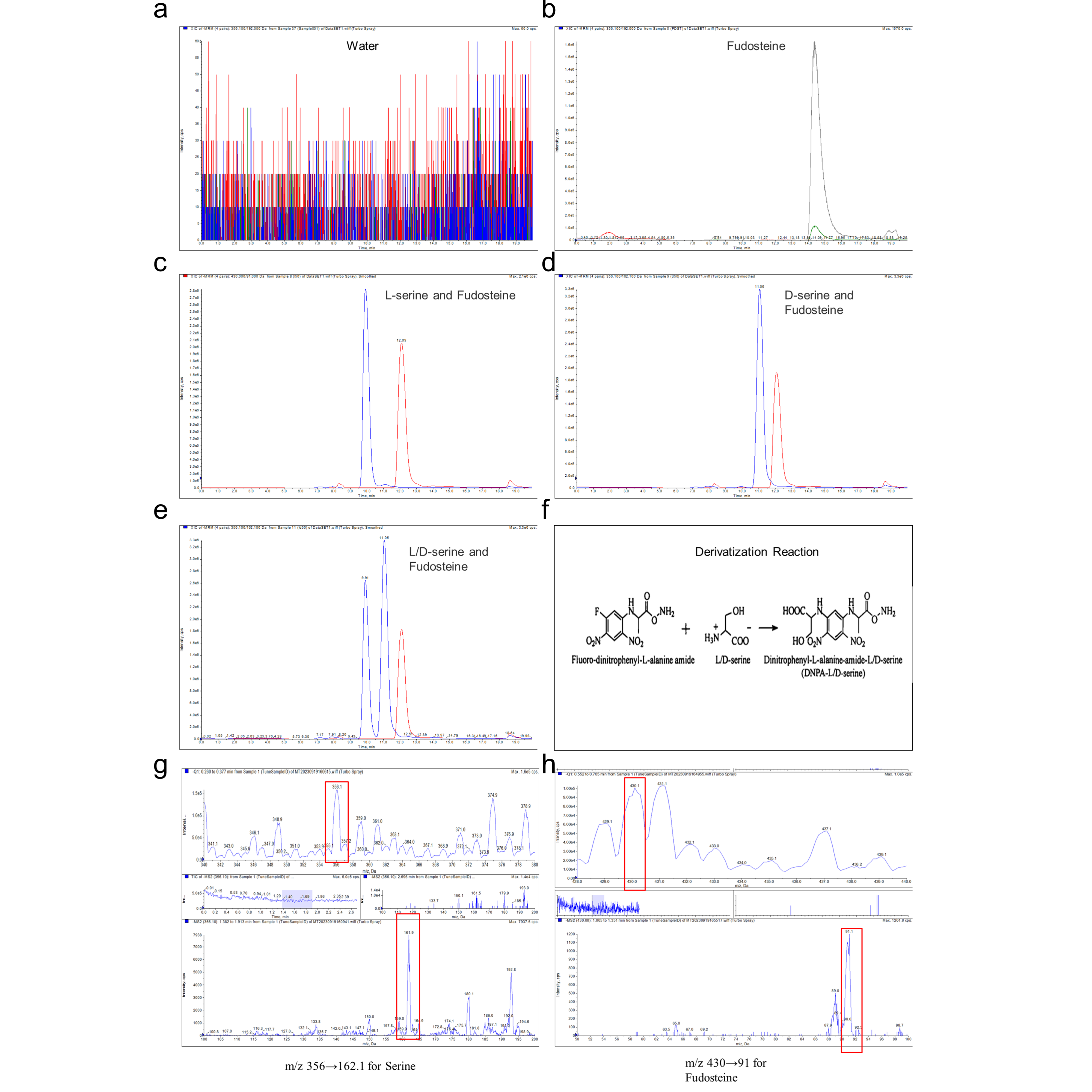


**Supplementary Fig. 3 Representative LC-MS/MS chromatograms for D-serine in water and multiple MRM transitions extracted ion chromatograms.** A, chromatogram of the water; B, chromatogram of the water with the standard 50 μg/mL fudosteine; C, chromatogram of the water with the standard 50 μg/mL fudosteine and 10 μg/mL L-Serine; D, chromatogram of the water with the standard 50 μg/mL fudosteine and 10 μg/mL D-Serine; E, chromatogram of the water with the standard 50 μg/mL fudosteine, 10 μg/mL D-Serine and 10 μg/mL F, the reaction equation of Marfey’s reagent with serine. G, MRM transitions extracted ion chromatograms for serine. H, MRM transitions extracted ion chromatograms for fudosteine.

**
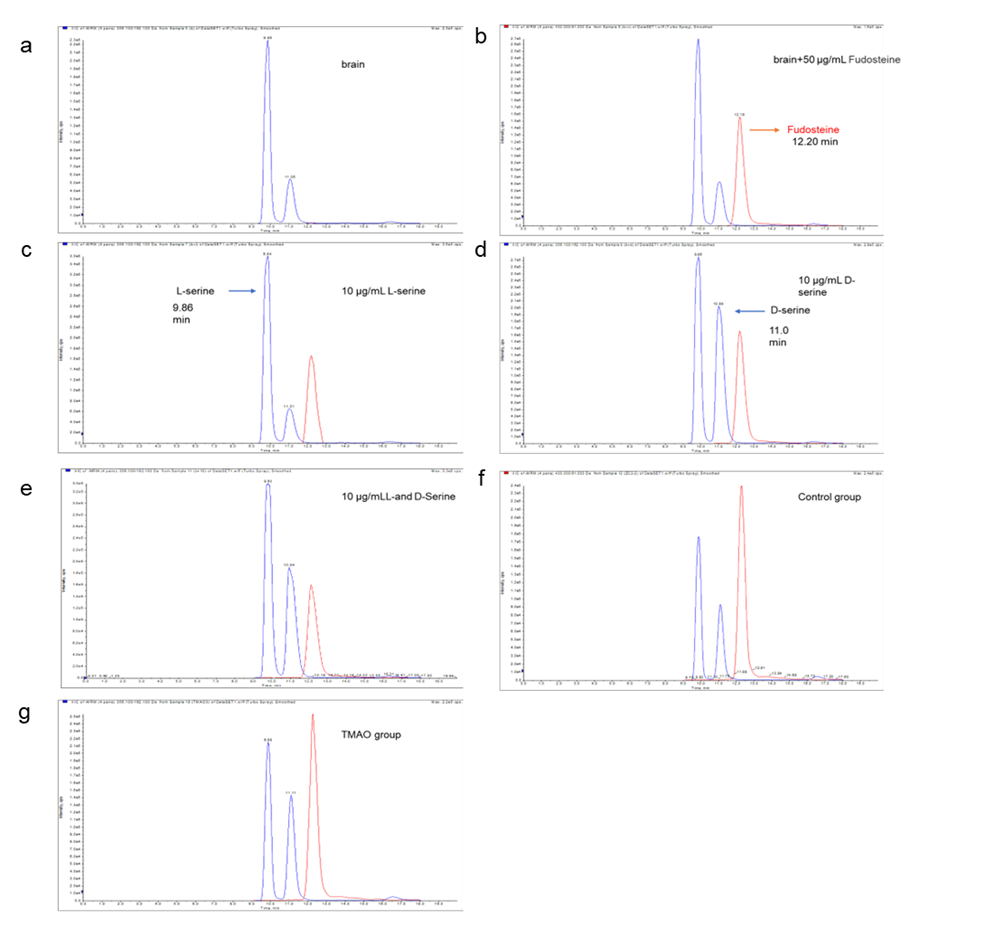
**

**Supplementary Fig. 4 Representative LC-MS/MS chromatograms for D-serine in brain.** A, chromatogram of the brain sample; B, chromatogram of the brain sample with the standard 50 μg/mL fudosteine; C, chromatogram of the brain sample with the standard 50 μg/mL fudosteine and 10 μg/mL L-Serine; D, chromatogram of the blank brain sample with the standard 50 μg/mL fudosteine and 10 μg/mL D-Serine; E, chromatogram of the blank brain sample with the standard 50 μg/mL fudosteine, 10 μg/mL D-Serine and 10 μg/mL D-Serine. F, chromatogram of the brain sample in control group; G, chromatogram of the brain sample in TMAO fed group. TMAO, trimethylamine N-oxide. Blue lines represented Serine and red lines represented fudosteine.


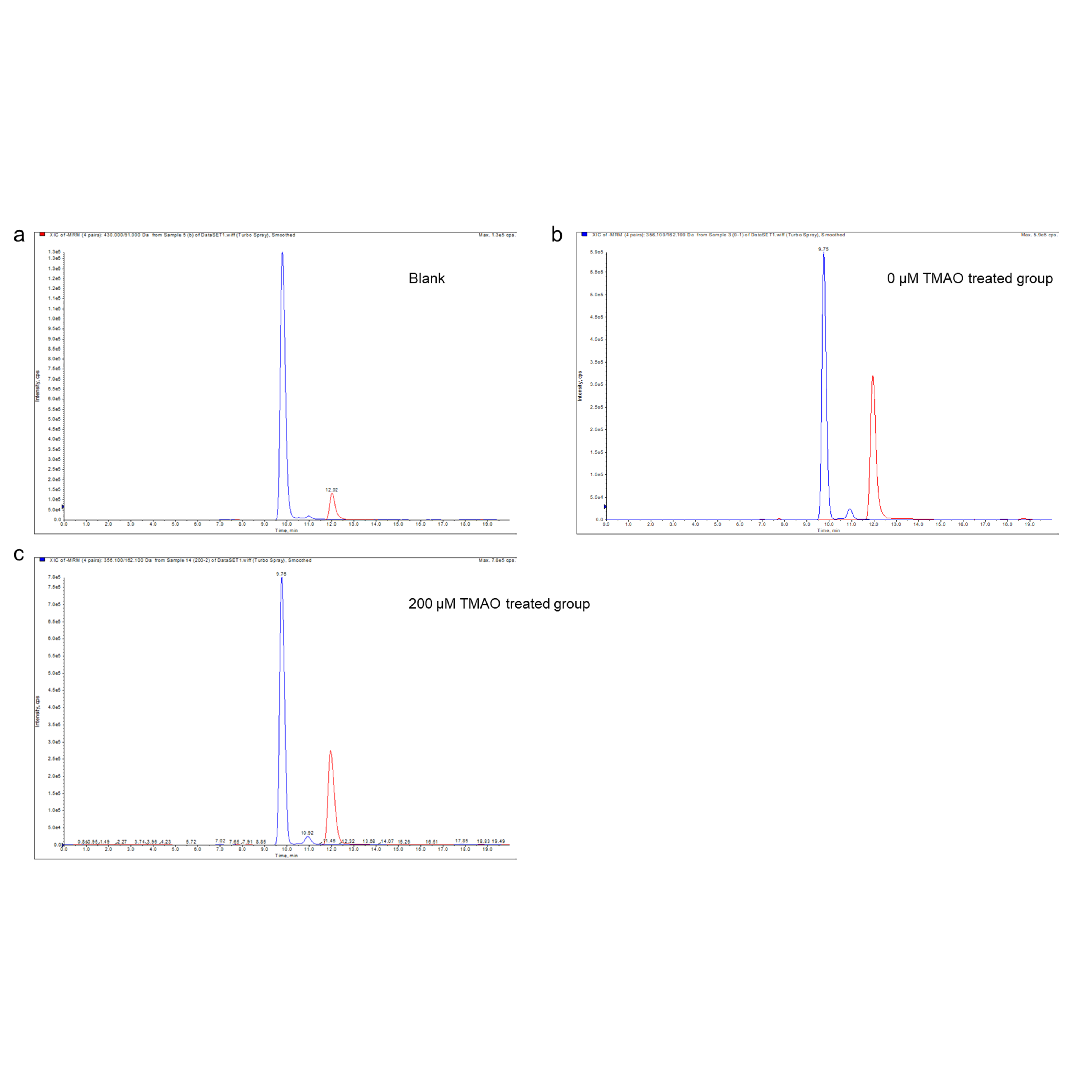


**Supplementary Fig. 5 Representative LC-MS/MS chromatograms for D-serine in DMEM.** A, chromatogram of the blank DMEM sample; B, chromatogram of the DMEM sample in 0 μmol/L TMAO treated group; C chromatogram of the DMEM sample in 200 μmol/L TMAO treated group. TMAO, trimethylamine N-oxide. Blue lines represented Serine and red lines represented fudosteine.

c


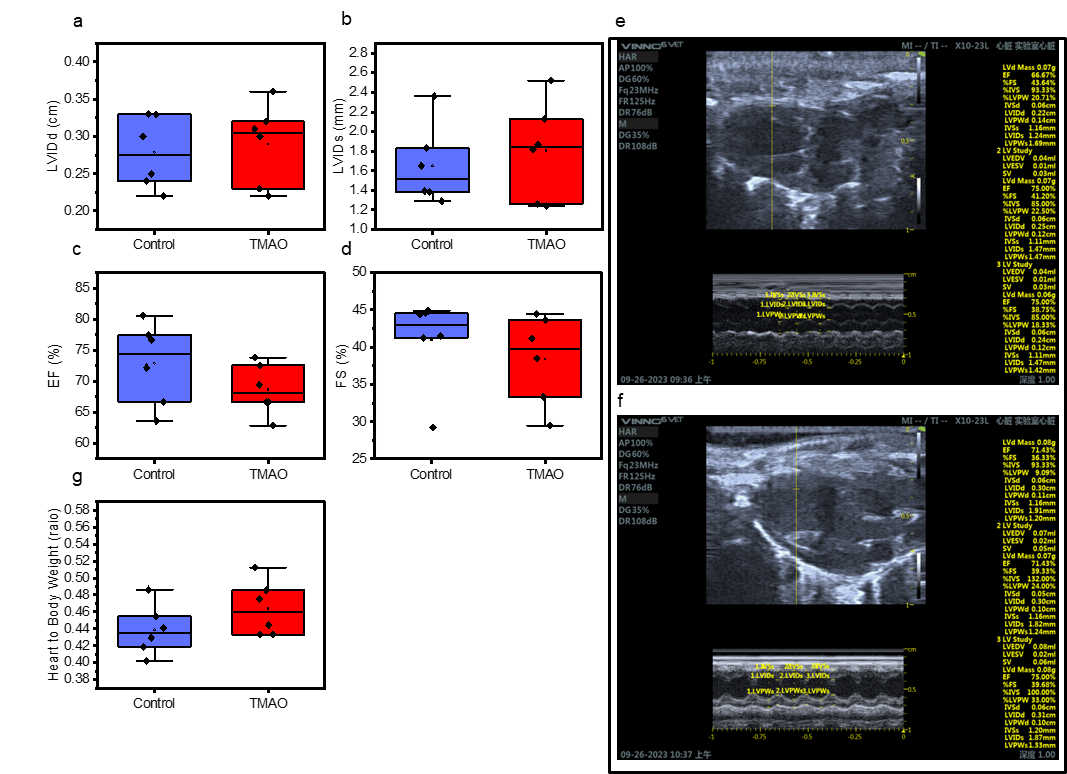


**Supplementary Fig. 6** **Echocardiography and cardiac indicators between two groups.** a, Echocardiographic analysis of left ventricular internal diameter at end-diastole (LVIDd). b, Echocardiographic analysis of left ventricular volume during systole (LVIDs). c, Echocardiographic analysis of left ventricular ejection fraction (LVEF). d. Echocardiographic analysis of left ventricular fractional shortening (LVFS). e. Representative echocardiographic analysis of control group. f. Representative echocardiographic analysis of TMAO group. g. Heart mass to body weight ratio.
